# The Taxonomic Status of *Mazama bricenii* and the Significance of the Táchira Depression for Mammalian Endemism in the Cordillera de Mérida, Venezuela

**DOI:** 10.1371/journal.pone.0129113

**Published:** 2015-06-29

**Authors:** Eliécer E. Gutiérrez, Jesús E. Maldonado, Aleksandar Radosavljevic, Jesús Molinari, Bruce D. Patterson, Juan M. Martínez-C., Amy R. Rutter, Melissa T. R. Hawkins, Franger J. Garcia, Kristofer M. Helgen

**Affiliations:** 1 Division of Mammals, National Museum of Natural History, Smithsonian Institution, Washington DC, United States of America; 2 Center for Conservation and Evolutionary Genetics, Smithsonian Conservation Biology Institute, National Zoological Park, Washington DC, United States of America; 3 Plant Biology and Conservation, Northwestern University, Evanston, Illinois, United States of America; 4 Department of Botany, National Museum of Natural History, Smithsonian Institution, Washington DC, United States of America; 5 Departamento de Biología, Facultad de Ciencias, Universidad de Los Andes, Mérida, Venezuela; 6 Integrative Research Center, Field Museum of Natural History, Chicago, Illinois, United States of America; 7 Instituto de Biología, Grupo Mastozoología & Colección Teriológica, Universidad de Antioquia, Medellín, Colombia; 8 Department of Ecosystem Science and Management, Pennsylvania State University, University Park, Pennsylvania, United States of America; 9 Department of Environmental Science & Policy, George Mason University, Fairfax, Virginia, United States of America; 10 Laboratorio Museo de Zoología, Departamento de Biología, Universidad de Carabobo, Valencia, Carabobo, Venezuela; Sichuan University, CHINA

## Abstract

We studied the taxonomy and biogeography of *Mazama bricenii*, a brocket deer classified as Vulnerable by the IUCN, drawing on qualitative and quantitative morphology and sequences of the mitochondrial cytochrome-*b* gene. We used Ecological Niche Modeling (ENM) to evaluate the hypothesis that *M*. *bricenii* of the Venezuelan Cordillera de Mérida (CM) might have become isolated from populations of its putative sister species, *Mazama rufina*, in the Colombian Cordillera Oriental (CO). This hypothesis assumes that warm, dry climatic conditions in the Táchira Depression were unsuitable for the species. Our analyses did not reveal morphological differences between specimens geographically attributable to *M*. *bricenii* and *M*. *rufina*, and phylogenetic analyses of molecular data recovered *M*. *bricenii* nested within the diversity of *M*. *rufina*. These results indicate that *M*. *bricenii* should be regarded as a junior synonym of *M*. *rufina*. ENM analyses revealed the existence of suitable climatic conditions for *M*. *rufina* in the Táchira Depression during the last glacial maximum and even at present, suggesting that gene flow between populations in the CO and CM may have occurred until at least the beginning of the current interglacial period and may continue today. Because this pattern might characterize other mammals currently considered endemic to the CM, we examined which of these species match two criteria that we propose herein to estimate if they can be regarded as endemic to the CM with confidence: (1) that morphological or molecular evidence exists indicating that the putative endemic taxon is distinctive from congeneric populations in the CO; and (2) that the putative endemic taxon is restricted to either cloud forest or páramo, or both. Only *Aepeomys reigi*, *Cryptotis meridensis*, and *Nasuella meridensis* matched both criteria; hence, additional research is necessary to assess the true taxonomic status and distribution of the remaining species thought to be CM endemics.

## Introduction

Deer of the northern Andes are among the least studied groups of medium- to large-sized mammals worldwide. They include members of the traditionally recognized but polyphyletic genus *Mazama* [1, 2, 3], as well as the genera *Odocoileus* [4] and *Pudu* [5]. The phylogenetic affinities, biogeography, ecology, behavior and even taxonomy of these deer are poorly understood. The Briceño’s brocket deer, *Mazama bricenii*, represents one of the best examples of this confusion. Oldfield Thomas [6] named the species after Salomón Briceño Gabaldón, a professional collector who provided him with specimens from the Venezuelan Andes, resulting in the description of several new species of mammals [7], including the single specimen of *M*. *bricenii* available for its description. This specimen was collected in the Páramo La Culata, in the Venezuelan Cordillera de Mérida (hereafter referred to as “CM”). This locality is situated at an elevation of 3000 m and is separated from the Cordillera Oriental of Colombia (hereafter referred to as “CO”) by the Táchira Depression, a relatively low (maximum elevation, 960 m), warm, and dry corridor [8]. In the original description, Thomas noted that while among the species of *Mazama* recognized at that time, *M*. *bricenii* was “undoubtedly most nearly allied to *Mazama tema*” from Central America (= *Mazama temama*; [9, 10, 11]), another skull from Ecuador more closely reassembled *M*. *bricenii*. Thomas mentioned that this similarity with the Ecuadorian specimen “indicates the existence in Ecuador of a highland Brocket allied to, and perhaps identical with, the animal now described from Venezuela” (= *M*. *bricenii*). This specimen from Ecuador was likely a *Mazama rufina*, which is the species of brocket that is currently known to occur in the Ecuadorean Andes [12]. Since the description of *M*. *bricenii*, most authors have treated it as a valid species, e.g., [13, 14, 15, 16,17], but some have regarded it either as a subspecies of *Mazama rufina*, e.g., [18, 19, 20, 21], or as a junior synonym of *M*. *rufina* [22]. This discrepancy is not surprising, because no modern taxonomic work has been carried out on Andean *Mazama*. In addition, the geographic isolation of the CM—to which some authors consider *M*. *bricenii* endemic—might have promoted the notion that populations from this cordillera are differentiated enough from those in the Andes of Colombia, Ecuador, and Peru, to merit taxonomic recognition at the species level. In fact, the Táchira Depression has been postulated as a barrier that could have isolated and promoted the differentiation of members of the fauna and flora endemic to higher, cooler, and mesic environments of the CM, e.g., [23, 24, 25, 26, 27, 28].

Regardless of the taxonomic status of *Mazama bricenii*, information about its distribution is equivocal. Whereas some authors have assumed that *M*. *bricenii* is endemic to the Venezuelan Andes [15, 20, 29, 30, 31, 32, 33], others have assumed that the species’ range also includes the CO of Colombia, where it has not been definitively verified [16, 17, 19, 34, 35, 36]. Despite this uncertainty, *M*. *bricenii* has been included in the most recent list of mammals present in Colombia [37], whereas it was regarded as a Venezuelan endemic in the most recent list of Venezuelan mammals [33]. Assertions about the presence of *M*. *bricenii* in Colombia have not been supported by publicly available, verifiable evidence; however, it is plausible that this deer is present in the Colombian Andes. Dietrich [34] reported a vouchered record from Páramo del Tamá, which, although located within Venezuelan political borders, it is part of the CO of Colombia. Linares [38] recorded the species in the Serranía de Perijá, northwestern Venezuela, close to Colombian territory (i.e., the west versant of the sierra); however, it is unclear whether voucher specimens were deposited in a zoological collection. Critically, all of the aforementioned alleged records of *M*. *bricenii* for either the proximity to Colombian territory or for Colombia itself should have relied on information that allows unambiguous distinction between *M*. *bricenii* and *M*. *rufina*, however, such information has never been published.

**Fig 1:**
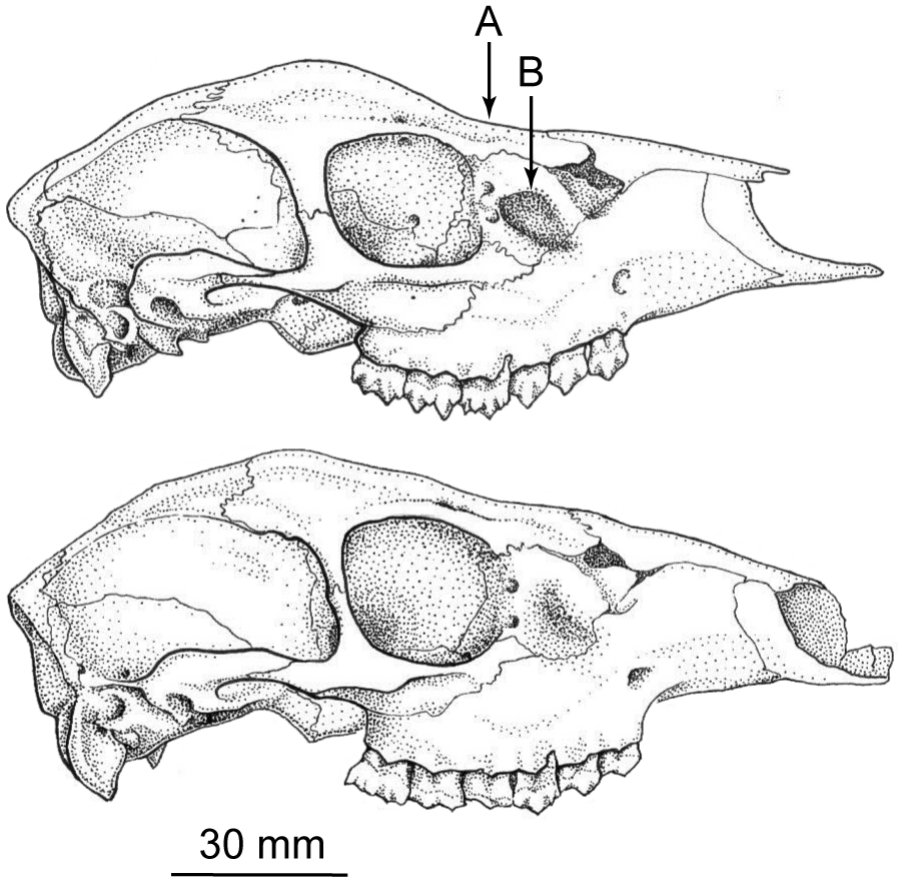
Possible qualitative cranial differences suggested from preliminary comparisons of topotypes of *Mazama bricenii* and *M*. *rufina*. Top: topotype of *Mazama bricenii* from La Culata, Mérida, Venezuela (FMNH 20197); bottom: topotype of *M*. *rufina* from Volcán Pichincha, Pichincha, Ecuador (FMNH 44335). Both specimens are adult females. (A) Lacrimal fossa: narrower and substantially deeper in the specimen of *M*. *bricenii* than in that of *M*. *rufina*; (B) Frontal bones: slightly depressed anteriorly in the specimen of *M*. *bricenii*, but relatively straight in that of *M*. *rufina*. Illustrations by Megan Krol.

**Fig 2:**
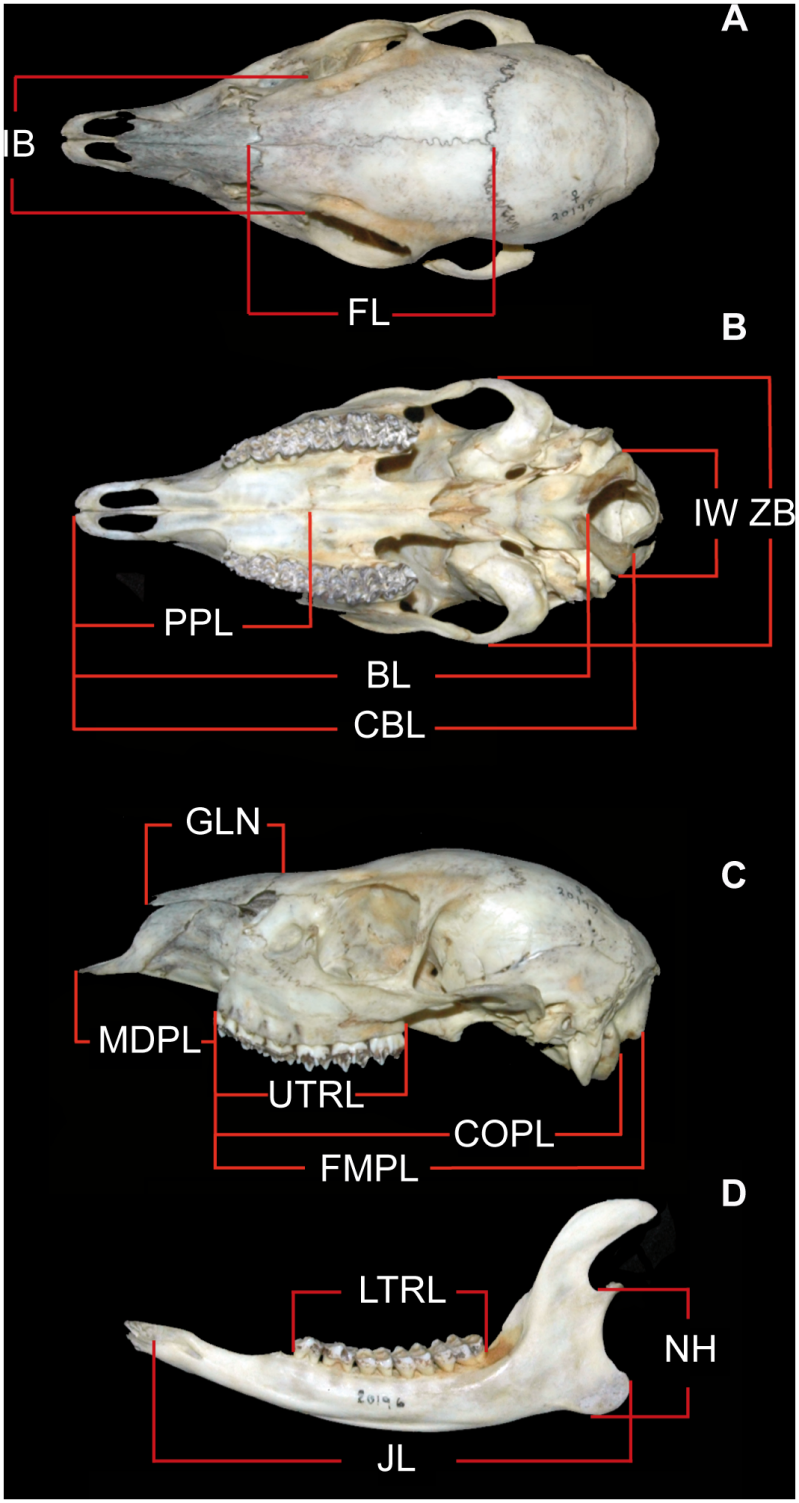
Linear measurements used in descriptive, univariate, and multivariate statistics. See Materials and Methods for names and descriptions of measurements.

Because proper assessments of taxonomic status of populations are essential for efficient conservation planning, e.g., [39, 40], and because firm documentation of a species’ presence within national borders is needed for countries to grant species protection, in the present study, we assess both of these aspects (taxonomy and extent of geographic distribution) for *Mazama bricenii*. This species is substantially different in pelage, skull morphology, overall body size, and geographic and ecological distribution to all other members of the genus *Mazama* (as currently understood) except *M*. *rufina* [5, 17, 41]. Consequently, we focused in comparisons and analyses of morphological and molecular data between *M*. *bricenii* and *M*. *rufina*. We first tested the validity of differences in qualitative cranial traits that we observed in preliminary side-by-side comparisons between topotypes of *M*. *bricenii* and *M*. *rufina* (Fig 1). We determined if these are consistent distinctions that hold taxonomic value across a larger number of specimens and a broader geographic sample (Fig 2). We then conducted both linear morphometric and molecular phylogenetic analyses (mtDNA) to assess the degree of distinctiveness of populations considered by various authors to correspond to *M*. *bricenii*. Finally, we employed Ecological Niche Modeling (ENM) analyses based on occurrence records and bioclimatic variables to test whether the Táchira Depression likely represented a barrier isolating *Mazama* populations in the CM. In addition to clarifying taxonomic boundaries within *Mazama*, our results prompted us to review the list of mammals currently considered endemic to the CM, and to propose criteria to evaluate putative cases of endemism in the CM.

## Materials and Methods

### Source of data

We examined and measured specimens housed in both North and South American collections (S1 File). Geographic data associated with these specimens were pooled together with information from the literature [34, 42] and used for Ecological Niche Modeling (ENM) analyses. The molecular data consisted of eleven sequences of the mitochondrial cytochrome-*b* gene (CYTB; 1140 bp). Six of them were obtained from degraded DNA extracted from museum specimens, from residual soft tissue attached to skeletons, or from maxilloturbinate bones [43]. For GenBank accession numbers and specimen’s information, see S1 File. The remaining sequences were downloaded from GenBank (accession numbers: JN632657, JN632671, NC020719, NC020721, NC020739). One of these sequences (NC020739) was mistakenly attributed to *Pudu mephistophiles* by Hassanin et al. [3]; comparisons of this sequence with those obtained from museum specimens of both *Pudu mephistophiles* and *M*. *rufina* allowed us to reidentify the species to which the sequence corresponds as *M*. *rufina* (page 40 of [3]) recognized the possibility that their identification was incorrect).

### Laboratory methods

In order to avoid contamination from exogenous DNA, we conducted DNA extractions and preparation of reactions previous to PCR amplification in an isolated ancient DNA laboratory located in a separate building from the one containing the primary DNA laboratory and where PCR products of high molecular weight mammalian DNA have never been present. For DNA extractions, we used the method described by Wisely et al. [43], and subsequently employed various combinations of primers to amplify and to sequence short CYTB fragments (S2 File. **Primer pairs used for amplification and sequencing of the CYTB gene**). These reactions were performed in a six-stage touchdown protocol using a thermal cycler (MJ Research). After an incubation at 95°C for 10 min, the first stage consisted of 2 cycles of the following steps: denaturing at 95°C for 15 seconds, annealing at 60°C for 30 seconds, and extension at 72°C for 1 min. The second, third, fourth, and fifth stages were identical to the first except for lowered annealing temperatures of 58°C, 56°C, 54°C, and 52°C, respectively. The final stage consisted of 40 cycles with an annealing temperature of 50°C. We performed the PCR in 25 μl volumes containing 0.5 U AmpliTaq Polymerase (Applied Biosystems, Foster City, CA), 1X PCR AmpliTaq Buffer, 0.2 μM each dNTP, 0.4 μM of forward and 0.4 μM of reverse primers, 1.5 μM MgCl2, 10X BSA (New England Biolabs, Ipswich, MA), and 50–250 ng of genomic DNA template Successful amplifications were purified using ExoSAP (USB Corporation, Cleveland, OH) incubated at 37°C for 15 min followed by 80°C for 15 min. Both strands of each PCR product were cycle sequenced by subjecting them to a second amplification using a total of 10 μL sequencing reaction mixture, including 50–200 ng of PCR product, 10 pM of corresponding forward or reverse primer, 5X Big Dye Buffer (Applied Biosystems), 1/8 reaction of Big Dye version 3 (Applied Biosystems). The following conditions were used for the Dye Terminator Cycle Sequencing: 25 cycles consisting of denaturing at 96°C for 10 s, annealing at 50°C for 10 s and extension at 60°C for 4 min. These final products were cleaned using Sephadex filtration and then both the 3’ and 5’ strands were sequenced on a 50 cm array using the ABI PRISM 3130 Genetic Analyzer (Applied Biosystems). We employed Geneious v.7.1.5. (Biomatters; http://www.geneious.com/) to compile and edit the sequences that we generated.

### Morphological comparisons and morphometric analyses

Specimens used in this study match characteristics that authors have employed to distinguish *Mazama rufina* and *M*. *bricenii* from other brockets, including the presence of a deep lacrimal fossa, black lower legs, a black mask on the head extending from the nose to the nape, and small skull and body size, among others [13, 16, 17, 34, 42]. However, because no information has been published (not even in their original taxonomic descriptions [6, 44] on how to distinguish *M*. *rufina* and *M*. *bricenii*, we had no option other than assigning species membership to each specimen based on geography (based on previous researchers’ assertions on the species distributions).

In a total of 22 specimens, we scored the state of two qualitative cranial characters observed in preliminary side-by-side comparisons between topotypes of *Mazama bricenii* (FMNH 20197) and *M*. *rufina* (FMNH 44335). These two traits were the relative depth of the lacrimal fossa—shallow in the topotype of *M*. *rufina*, but deeply depressed in the specimen of *M*. *bricenii*—and the shape of the frontals—relatively straight in the topotype of *M*. *rufina*, but depressed anteriorly in the specimen of *M*. *bricenii* (Fig 1).

Our morphometric analyses were based on 14 linear measurements of the cranium and mandible, described as follows (Fig 2): *interorbital breadth* (IB), maximum width across the anterior extremes of the orbits (this measurement is taken with the caliper tips right below the orifice of the lacrimal duct of each orbit); *frontal length* (FL), length of the frontal at the midline of the skull, taken from the suture formed by the frontal and the nasal (i.e. anteriormost point of the frontal) to the suture of the frontal and the parietal (i.e. anteriormost point of the parietal); *intercondylar width* (IW), maximum distance between the external borders of the occipital condyles; *zygomatic breadth* (ZB), greatest distance between the outer margins of the squamosal arms of the zygomatic arches; *palatine-premaxillary length* (PPL); length from the suture between the palatine and the maxilla (i.e., anteriormost point of the palatine) to the anteriormost point of the premaxilla; *basal length* (BL), length from the anteriormost point on the lower border of the foramen magnum to the anteriormost point of the premaxilla; *condylobasal length* (CBL), length from the posteriormost point of the occipital condyles to the anteriormost point of the premaxilla; *greatest length of nasals* (GLN), length of the nasals at the midline of the skull, taken from the suture formed by the frontal and the nasals (i.e. anteriormost point of the frontal) to the anteriormost point of the nasal bones; *maxillary diastema-premaxillary length* (MDPL), distance from the anteriormost point of the second upper premolar to the anteriormost point of the premaxilla; *upper tooth row length* (UTRL), distance from the anteriormost point of the second upper premolar to the posteriormost point of the third upper molar; *occipital condyle-premolar length* (COPL), length from the posteriormost point of the occipital condyles to the anteriormost point of the second upper premolar; *lower tooth row length* (LTRL), distance from the anteriormost point of the second lower premolar to the posteriormost point of the third lower molar; *notch height* (NH), maximum distance from the lowest point of the notch of the mandible to the most ventral point of the angle; and *jaw length* (JL), maximum distance of the mandible from the most lateral point of the alveolar margin of the canine socket to the posteriormost point of the angle. Measurements were taken to the nearest 0.01 mm with digital calipers.

**Fig 3:**
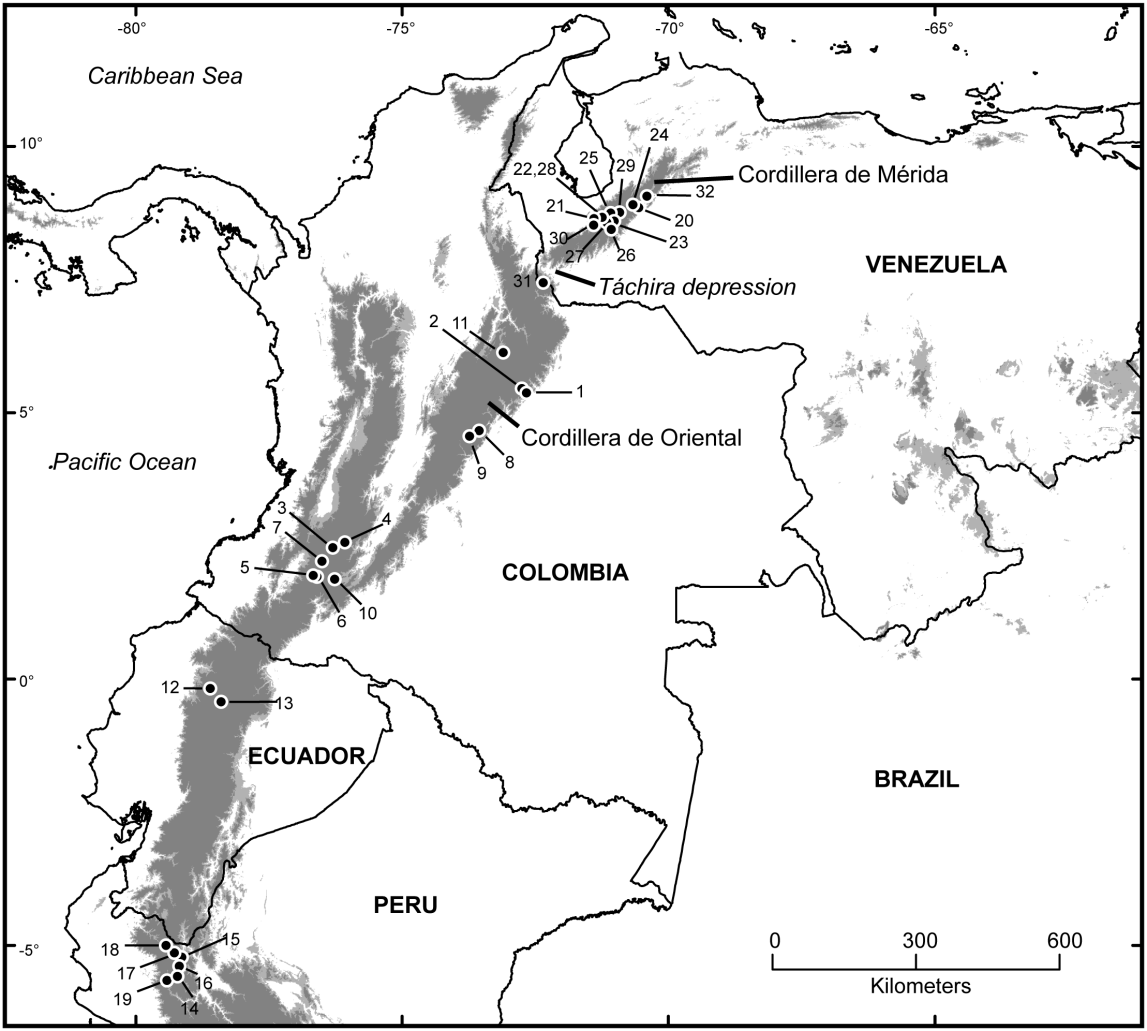
Map of *Mazama* localities. Circles represent localities; numbers correspond to entries in S1 File. Progressively darker shading indicates areas with elevations of 1000–1500 m (pale gray) and above 1500 m (dark gray). Localities numbers 12 and 25 represent the type localities of *Mazama rufina* and *M*. *bricenii*, respectively.

We conducted univariate and multivariate analyses to assess possible secondary sexual dimorphism and differences among geographic groups. These analyses and descriptive statistics were based only on adult specimens (those with complete permanent dentition); two specimens (CVULA I-2657, I-8559) with marked toothwear, a sign of advanced age, were excluded from the analyses. To evaluate the extent of sexual size dimorphism, we compared the means of each measurement via a 2-tailed two-sample *t* tests performed in the R Language and Environment for Statistical Computing (hereafter referred to as “R”) [45] with the function *t*.*test*. This test assumes that the data are normally distributed; Shapiro-Wilks normality tests conducted for each measurement (with R´s native function *shapiro*.*test*) could not reject the null hypothesis of data being normally distributed. For multiple-comparison corrections, we used the simple and conservative Bonferroni correction (hereafter “BoC”) [46] and the more liberal sequential correction proposed by Benjamini and Hochberg [47] (hereafter “BHC”), both calculated with the R package “*stats”* (function *p*.*adjust*) [45]. We carried out two sets of comparisons; the first was based only on data taken from specimens from the CM (6 ♀♀, 2 ♂♂), whereas the second was based on data from all specimens (15 ♀♀, 6 ♂♂). Failure to detect significant differences between sexes (all BoC *p* values ≥ 0.447; all BHC *p* values ≥ 0.285) allowed us to use data from both sexes pooled together in subsequent analyses, thus increasing statistical power for these analyses. The latter consisted of three sets of comparisons between measurement means of geographically defined groups, as follows: (1) a comparison between the two putative species based on data from specimens from either the type locality or nearby sites (hereafter referred to as “regional topotypes”) of both species—i.e. specimens of *M*. *bricenii* from the CM (*n* = 7) vs. Ecuadorian specimens of *M*. *rufina* from the Pichincha and Cotopaxi mountains (*n* = 4); (2) a comparison between specimens to the east (putatively representing *M*. *bricenii; n =* 7) and west (*M*. *rufina*; *n* = 14) of the Táchira Depression (see Fig 3 for geographic references)—this comparison aimed to discover differences aligned with the hypothesis that the Táchira Depression promoted morphological differentiation in populations to the east of that depression via geographic isolation; (3) a comparison between a group formed by Venezuelan specimens plus Colombian specimens from the CO (*n* = 13) vs. a group formed by Colombian specimens from the Cordilleras Central and Occidental plus Ecuadorian specimens (*n* = 8)—this comparison aimed to uncover differences between samples from regions from which previous authors have assigned specimens to either *M*. *bricenii* or *M*. *rufina* (see Introduction). Descriptive statistics for geographic groups were calculated with the R package *psych* [48].

We conducted a principal component analysis (PCA) to detect possible morphometric discontinuities among geographic groups. Because multivariate analyses require complete datasets, we estimated missing values (those lacking due to cranial or mandibular damage) with the Probabilistic Principal Component Analysis method as implemented in the R package *pcaMethods* [49]. After transforming measurement values into natural logarithms, we conducted the PCA based on the correlation matrix using the Paleontological Statistics Software Package for Education and Data Analysis (PAST ver. 3.02) [50].

### Phylogenetic analyses and genetic distances

Before conducting phylogenetic analyses, we checked the quality of each DNA sequence, aligned them, and employed the resulting matrix to determine both the most suitable partition scheme and the best fitting model of nucleotide substitutions (for each data subset, if any). All sequences were translated to amino acid sequences using Geneious ver. 7.1.5 and examined to assure that no premature stop codons were present. Subsequently, sequences were aligned using default options in MAFFT ver. 7.017 [51] as implemented in Geneious. Partition schemes and model of nucleotide substitution for each data subsets were selected with PartitionFinder ver. 1.0.1 [52] using the corrected Bayesian Information Criterion (BIC) and considering models applicable in MrBayes [53].

We conducted phylogenetic analyses using maximum likelihood (ML) and Bayesian inference (BI) optimality criteria. For all analyses, we used one sequence of *Odocoileus cariacou* (name follows the provisional taxonomic arrangement for *Odocoileus* proposed by Molina and Molinari [4], and Molinari [25]) and two of *Mazama americana* as outgroups (GenBank accession numbers JN632671, NC020719, JN632657). According to a previous study, these two species are closely related to *M*. *rufina* [3]. The ML analysis was conducted with 20 independent searches in the Genetic Algorithm for Rapid Likelihood Inference (GARLI 2.0) [54] using the default settings. The Bayesian inference analysis was conducted in MrBayes v. 3. 2 [53]. The search started with a random tree, and the Markov chains were run for 100,000,000 generations; trees were sampled every 1000 generations. Default values were kept for the ‘‘relburnin” and ‘‘burninfrac” options in MrBayes (i.e. relburnin = yes; burninfrac = 0.25); therefore, the first 25,000,000 generations (25,000 trees) were discarded as burn-in. To assess nodal support, posterior probability estimates were obtained based on the remaining (75,000) trees. For the ML analysis, nodal support assessed with nonparametric bootstrapping [55]. This analysis was also conducted with GARLI and was based on 1000 searches (100 pseudoreplicated data matrices, and 10 searches for each of them). The degree of support received by individual nodes in the ML bootstrap analysis was categorized as follows: *strong* if bootstrap value ≥ 75%; *moderate* if bootstrap value > 50% and < 75%; *negligible* if value ≤ 50%. For the BI analysis, the categorization of nodal support is as follows: *strong* if posterior probability ≥ 0.95; *negligible support* if posterior probability values < 0.95.

A high degree of sequence divergence is neither necessary nor sufficient for species recognition [56, 57, 58]; however, pairwise genetic distances provide a heuristic basis for comparisons of genetic variation within and among lineages [59]. Thus, we calculated the average uncorrected (*p*) distance within each haplogroup and the average pairwise *p* distances among them. We also report the commonly used Kimura 2-parameter-corrected distances to facilitate comparisons with data from the literature for non-volant, terrestrial mammals. Genetic distances were calculated using MEGA ver. 5.2.1 [60].

### Ecological Niche Modeling

We constructed Ecological Niche Models (ENMs) using the maximum entropy algorithm Maxent [61] to assess whether the Táchira Depression represents a current or past climatic barrier to dispersal. To accomplish this, we trained preliminary models using an appropriate study region (see below) and then projected them onto a larger study region twice, once while employing current climatic conditions and the second time using climatic conditions estimated for the Last Glacial Maximum (LGM).

Occurrence records were gathered from our own examination of museum specimens and from the literature [34, 42], and were georeferenced with maps, gazetteers, and digital resources (S1 File). Because we did not have *a priori* information regarding which occurrence records corresponded to *Mazama bricenii* and which to *M*. *rufina*, or, alternatively, if all records would actually belong to a single species, we first applied the methods described above to determine the taxonomic status of *M*. *bricenii*. To mitigate potential effects of sampling bias and spatial autocorrelation [62], we employed the R package spThin [63] to spatially filter occurrence records to have a minimum distance of at least 10 km among them (i.e. so that no record was closer than 10 km to any other record). After filtering, 22 occurrence records from unique localities remained for modeling.

The environmental data used to create the models consisted of 19 bioclimatic variables from WorldClim 1.4 [64] that previous studies found to be important in determining mammal species distributions, e.g., [65, 66, 67]. To define an appropriate study region for model calibration, we followed the criteria proposed by Anderson and Raza [65], and the operational strategy used by Gutiérrez et al. [67]. Thus, we employed ArcGIS 10.2 to first create a minimum convex hull that enclosed the filtered occurrence records, and then buffered this at a distance of 50 km. As mentioned above, the models were then projected to a larger study region (extend: 7.0° S–13.5° N and 60.0° W–81.5° W) under both current climatic conditions and also using estimated paleoclimatic data for the LGM from two general circulation models (GCMs): the Community Climate System Model (CCSM4) [68] and the Model for Interdisciplinary Research on Climate (MIROC-ESM) [69]. The environmental dataset corresponding to present-day climatic conditions was at a resolution of 30 seconds, whereas those corresponding to LGM climatic conditions had coarser resolution of 2.5 minutes.

Ecological Niche Models were created using Maxent ver. 3.3.3h [61]. Since several recent studies have demonstrated the importance of selecting model Maxent settings carefully to balance model’s performance and complexity, we tuned the value of the regularization multiplier and determined the optimal selection of feature classes [66, 70, 71, 72]. All tuning experiments were implemented using the R package ENMeval [73]. The regularization multiplier was varied from 0.5 to 6.0 in increments of 0.5, and the following four feature classes (or combinations thereof) were tested: (1) linear; (2) linear and quadratic; (3) hinge; and (4) linear, quadratic, and hinge. ENMeval allows for several data-partitioning schemes; for this study we employed the “checkerboard1” approach, a variation on the ‘masked geographically structured’ data-partitioning strategy described in Radosavljevic and Anderson [71]. Model performance was assessed using the Akaike Information Criterion corrected for small sample sizes (AICc) [70, 72]. The final model was constructed with the combination of regularization multiplier and feature classes that yielded the lowest value of AICc, and employing all filtered, georeferenced occurrence records. As AICc can only select the ‘best’ model from among a set of models, and does not directly assess model performance, we also inspected omission rate and test AUC of the models selected as optimal. Additionally, since we were projecting these models onto a different region than that used for calibrating the model, we inspected the multivariate environmental similarity surfaces and clamping maps produced by Maxent to determine whether any environmental variables on the larger region were outside the range of climatic conditions present in the calibration study regions.

Final models were converted into binary maps of “suitable” vs “unsuitable” habitat using the minimum training presence threshold calculated by Maxent. This threshold considers as suitable all pixels that have a suitability score equal to or greater than the lowest suitability predicted for a known occurrence. While this threshold value is likely to include some habitat that may be marginally suitable (due to real and artifactual causes; [71, 74]), for our purposes it is an objective and logical choice—our goal is to test whether a warm and dry area (i.e., the Táchira Depression) acts as a barrier to dispersal, thus even marginal habitat should be identified.

## Results

### Morphological comparisons and morphometric analyses

Two differences in qualitative cranial traits observed between *Mazama bricenii* and *M*. *rufina* in initial comparisons that included topotypes of both species—i.e. depth of lacrimal fossa and shape of frontal bones (Fig 1)—proved to be extremely variable, and do not hold taxonomic value. In a total of 22 specimens examined, we found (a) deep lacrimal fossae and frontal bones posteriorly depressed in eight females, five from the CM, two from the CO, and one from Ecuador; (b) shallow lacrimal fossae and straight frontal bones in two females from Ecuador; (c) deep lacrimal fossae but straight frontal bones in three specimens, one female from CM and one male and one female from the Cordillera Central of Colombia. In addition, depth of the lacrimal fossa in two specimens was scored as intermediate between “deep” and “shallow” (see Fig 1), and the shape of the frontal bones in nine specimens was intermediate between “depressed” and “straight”. These observations do not reveal any consistent pattern of geographic variation.

We did not detect significant differences in size in comparisons between topotypes of *Mazama bricenii* and *M*. *rufina*, or in comparisons between specimens separated by the Táchira Depression (in all of these analyses BoC *p* values ≥ 0.469 and BHC *p* values ≥ 0.219). Except for two measurements, comparisons between a group formed by specimens from Venezuela and the CO of Colombia—where previous authors have reported deer allegedly identified as *M*. *bricenii*—as compared with a group formed by specimens of *M*. *rufina* from Ecuador and from the Cordillera Central of Colombia failed to detect significant differences (*p* values ≥ 0.116 for both BoC and BHC). However, the former group has significantly narrower skulls (mean of zygomatic arches = 71.06 mm; *p* values for both BoC and BHC for t-tests ≤ 0.032) and marginally significantly shorter jaws (mean of jaw length = 120.99 mm; *p* values for the BHC for t-tests = 0.070) than specimens from Ecuador and the Cordillera Central of Colombia (75.90 mm and 129.75 mm, respectively). Descriptive statistics for geographic groups are shown in Tables 1 and 2.

**Table 1:** Measurements of *Mazama* specimens from Venezuela and the Cordillera Oriental of Colombia.

	Venezuela	Venezuela	Venezuela	Colombia
	Cordillera de Mérida. ♂♂	Cordillera de Mérida. ♀♀	Páramo del Tama. ♀♀	Cordillera Oriental. ♀♀
IB	40.47 ± 2.92 (38.40–42.53), 2	37.00 ± 3.57 (33.65–42.82), 5	32.10, 1	40.59 ± 0.68 (39.73–41.49), 5
FL	66.09 ± 3.39 (63.69–68.48), 2	61.89 ± 4.59 (57.57–67.07), 5	59.80, 1	54.97 ± 5.28 (48.63–62.44), 5
IW	33.91 ± 1.02 (33.19–34.63), 2	32.24 ± 0.70 (31.32–33.01), 4	30.10, 1	31.61 ± 1.61 (29.33–32.97), 5
ZB	72.84 ± 2.02 (71.41–74.26), 2	70.89 ± 1.62 (69.57–72.99), 4	71.10, 1	70.48 ± 4.54 (64.49–76.65), 5
PPL	64.66 ± 3.77 (62.00–67.33), 2	67.44 ± 5.27 (61.35–75.45), 5	68.50, 1	65.67, 1
BL	142.80 ± 9.28 (136.23–149.36), 2	138.61 ± 3.73 (133.05–140.84), 4	143.30, 1	139.44, 1
CBL	152.59 ± 9.58 (145.81–159.36), 2	149.74 ± 3.06 (145.48–152.35), 4	154.80, 1	149.96, 1
GLN	35.19 ± 5.20 (31.51–38.86), 2	41.86 ± 7.37 (35.25–54.09), 5	40.80, 1	42.42 ± 4.82 (38.33–47.73), 3
DLM	45.17 ± 5.18 (41.51–48.84), 2	48.96 ± 5.10 (41.66–55.34), 5	46.30, 1	47.07, 1
UTRL	50.49 ± 3.71 (47.87–53.12), 2	48.46 ± 2.86 (43.51–50.83), 5	49.90, 1	49.22 ± 3.14 (45.06–52.61), 5
COPL	112.95 ± 1.22 (112.09–113.82), 2	111.37 ± 4.20 (105.64–115.12), 4	108.60, 1	107.16 ± 5.54 (99.26–113.04), 5
LTRL	56.59 ± 0.82 (56.01–57.17), 2	54.37 ± 3.20 (48.95–57.46), 5	54.10, 1	53.78, 1
NH	33.41 ± 0.62 (32.98–33.85), 2	34.49 ± 3.24 (30.32–38.80), 5	33.40, 1	34.43, 1
JL	119.69 ± 7.59 (114.33–125.06), 2	121.53 ± 8.85 (113.20–136.56), 5	124.00, 1	117.86, 1

Descriptive statistics are: mean ± standard deviation (minimum–maximum), sample size. All measurements are expressed in millimeters. Names of measurements and their descriptions are provided in Materials and Methods and illustrated in Fig 2.

**Table 2:** Measurements of *Mazama* specimens from the Cordillera Central of Colombia and Ecuador.

	Colombia	Colombia	Andes of Ecuador	Andes of Ecuador
	Cordillera Central. ♂♂	Cordillera Central. ♀♀	♂♂	♀♀
IB	46.25, 1	35.31 ± 1.78 (34.05–36.57), 2	38.67 ± 5.51 (34.78–42.57), 2	42.59 ± 2.25 (41.29–45.19), 3
FL	61.82, 1	57.74, 1	60.28 ± 2.16 (58.76–61.81), 2	61.16 ± 4.11 (56.99–65.21), 3
IW	30.52, 1	31.27 ± 1.56 (30.17–32.37), 2	33.5 ± 0.70 (33.00–33.99), 2	31.99 ± 0.82 (31.36–32.92), 3
ZB	74.29, 1	75.86 ± 0.84 (75.27–76.46), 2	75.89 ± 0.60 (75.47–76.32), 2	76.46 ± 5.14 (71.53–81.79), 3
PPL	69.04, 1	67.34 ± 2.33 (65.69–68.98), 2	70.73 ± 1.41 (69.73–71.73), 2	70.2 ± 2.93 (68.37–73.58), 3
BL	149.76, 1	143.49 ± 6.96 (138.57–148.41), 2	145.38 ± 5.61 (141.41–149.34), 2	149.44 ± 8.89 (143.29–159.63), 3
CBL	161.78, 1	154.09 ± 6.36 (149.60–158.59), 2	157.91, 1	161.09 ± 9.54 (153.57–171.82), 3
GLN	40.56, 1	40.56, 1	48.20, 1	47.47 ± 4.01 (42.85–49.97), 3
DLM	52.05, 1	48.09 ± 1.28 (47.19–49.00), 2	52.39 ± 0.01 (52.38–52.40), 2	54.64 ± 6.33 (50.87–61.95), 3
UTRL	52.10, 1	52.59 ± 3.56 (50.08–55.11), 2	50.31 ± 0.09 (50.25–50.38), 2	47.89 ± 3.75 (43.61–50.59), 3
COPL	112.67, 1	104.86 ± 1.91 (103.51–106.21), 2	110.05 ± 2.18 (108.50–111.59), 2	108.39 ± 3.74 (104.70–112.17), 3
LTRL	57.47, 1	58.55 ± 5.98 (54.32–62.78), 2	54.31 ± 0.17 (54.19–54.43), 2	57.65 ± 2.16 (56.12–59.18), 2
NH	37.43, 1	34.71 ± 1.82 (33.42–36.00), 2	33.8 ± 1.42 (32.79–34.80), 2	35.03 ± 2.58 (33.20–36.85), 2
JL	127.39, 1	130.75 ± 3.66 (128.16–133.33), 2	132.27 ± 4.57 (129.04–135.50), 2	127.4 ± 4.00 (124.57–130.23), 2

Descriptive statistics are: mean ± standard deviation (minimum–maximum), sample size. All measurements are expressed in millimeters. Names of measurements and their descriptions are provided in Materials and Methods and illustrated in Fig 2.

**Fig 4:**
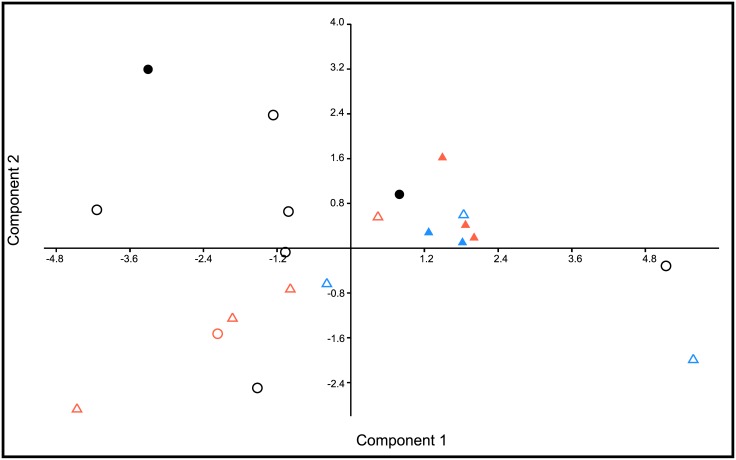
Plot of specimen scores on the first two axes of the principal components analysis of skull measurements of adult specimens of *Mazama*. Solid and open symbols represent male and female specimens, respectively. Geographic provenance represented as follows: black circles: Cordillera de Mérida (Venezuela); red circles: Cordillera Oriental (Colombia); red triangles: Cordillera Central (Colombia); blue triangles: Ecuador. PC1 is a size axis in which larger specimens appear toward the right side of the axis, whereas PC2 represents differences in cranial proportions (Table 3).

The principal components analysis did not reveal morphometric discontinuities among geographic groups. The first and second components explained 49.99% and 15.90% of the variance, respectively. Because the sign of the loadings of the first component (PC1) are all the same, we interpret PC1 as an axis that captured primarily differences in size among specimens (Table 3). Seven measurements (ZB, PPL, BL, CBL, GLN, DLM, JL) had the largest loadings in PC1. Some loadings on the second component (PC2) are negative and others positive; hence, we interpret PC2 as an axis that primarily captured differences in cranial proportions among specimens. In this component, the measurements with the largest loadings were GLN and DLM (both loading negatively) and UTRL and LTRL (both loading positively). A scatter plot constructed with specimens’ scores on the first two components shows substantial overlap among geographic groups in both components (Fig 4). Specimens from the CM, including the type locality of *Mazama bricenii*, are widely scattered across PC1 and PC2, overlapping with specimens from the Colombian and Ecuadorian Andes.

**Table 3:** Results of the principal component analysis based on measurements of specimens of *Mazama* from the northern Andes.

	PC 1	PC 2	PC 3	PC 4	PC 5	PC 6	PC 7
**IB**	0.16	-0.18	0.24	0.71	-0.14	-0.07	0.49
**FL**	0.18	0.35	0.43	-0.18	-0.12	-0.53	-0.15
**IW**	0.01	0.34	0.26	0.25	0.85	0.10	-0.02
**ZB**	0.34	0.13	-0.03	-0.07	0.01	0.12	-0.16
**PPL**	0.35	0.00	-0.07	-0.22	0.03	-0.02	0.26
**BL**	0.36	-0.01	0.08	0.06	-0.09	-0.14	-0.02
**CBL**	0.37	-0.01	0.06	-0.03	-0.06	-0.16	0.03
**GLN**	0.30	-0.15	-0.32	-0.15	0.29	0.03	0.02
**DLM**	0.33	-0.26	0.07	-0.07	0.11	0.03	0.21
**UTRL**	0.02	0.51	-0.43	0.12	-0.17	0.29	0.30
**COPL**	0.16	0.31	0.46	-0.21	-0.24	0.61	0.10
**LTRL**	0.12	0.49	-0.33	0.29	-0.12	-0.31	-0.14
**NH**	0.27	-0.16	-0.01	0.40	-0.13	0.31	-0.69
**JL**	0.34	-0.05	-0.25	-0.10	0.14	0.02	0.05
**Eigenvalue**	7.00	2.23	1.59	1.08	0.78	0.43	0.35
**% variance**	49.99	15.90	11.34	7.69	5.57	3.05	2.53

Components 8–14 represent less than 5% of total variance and are therefore omitted. Names of measurements and their descriptions are provided in Materials and Methods and illustrated in Fig 2.

### Molecular data and models of molecular evolution

Despite reliance on degraded DNA from museum specimens, our sequence matrix had only ca. 7% missing data (i.e., entries coded as unknown). PartitionFinder found the best partitioning scheme to be one formed by two subsets, one subset containing bases in the first and second codon positions, and another subset containing bases in the third codon position. The structure of the model for both subsets corresponds to the Hasegawa-Kishino-Yano model [75] with a proportion of invariant sites (i.e., HKY+I). The fact that PartitionFinder assessed that the best fit-model was the same for both subsets but still kept them separate means that the parameter values for these two subsets were sufficiently different that they were best modeled separately.

### Phylogenetic analyses and genetic distances

**Fig 5:**
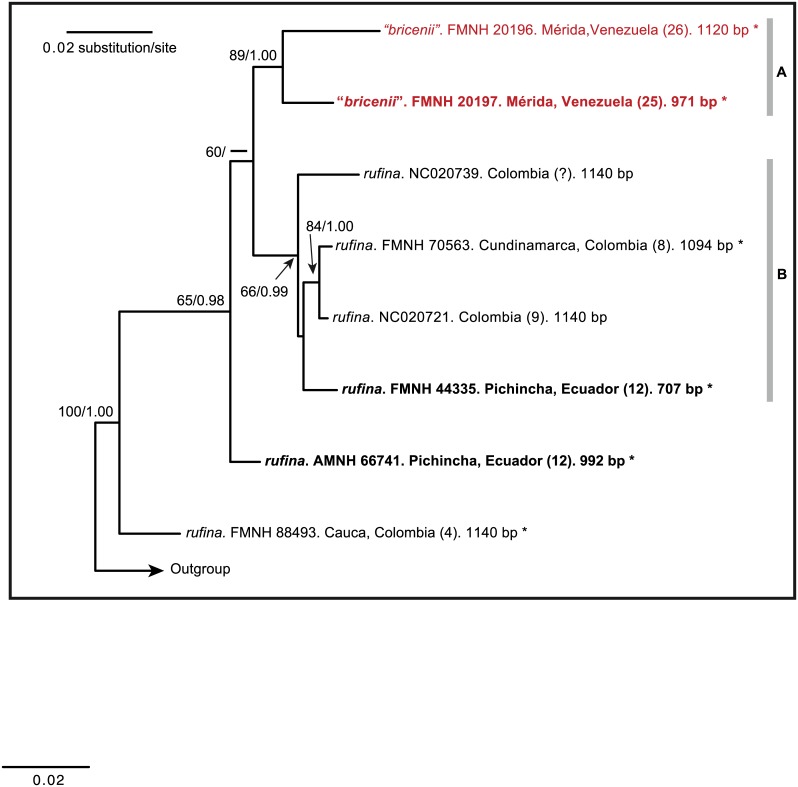
Phylogenetic tree of cytochrome-*b* sequences of *Mazama* from the northern Andes. This is the best topology resulting from the maximum-likelihood analysis. Nodal support is indicated at each node, except when the involved relationship received negligible support. Bootstrap values (from the maximum likelihood analysis) and posterior probabilities (from the Bayesian inference analysis) are indicated before and after the slash (“/”). Three topotypes (one *M*. “*bricenii*” and two *M*. *rufina*) are indicated with bold type (see detailed locality information in S1 File). The length of each sequence (number of base pairs, bp) is indicated at each terminal label. Asterisks denote sequences obtained from DNA extracted from museum specimens; all other sequences were downloaded from GenBank.

Our analyses of CYTB sequences did not uncover phylogeographic patterns nor did they establish the phylogenetic distinction of *Mazama bricenii*. After examining the average standard deviation of split of our BI analysis and confirming that it reached stationarity, we compared its topology with that resulting from the ML analysis. Both trees recovered the same branching patterns with nearly the same degree of nodal support. The two samples from the CM (one topotype and one near-topotype of *M*. *bricenii*) were recovered as sister to each other with strong nodal support (haplogroup A in Fig 5), but embedded within the diversity of *M*. *rufina*. Sister to this haplogroup (A) was another (B) formed by samples from unknown localities within Colombia (reported by Hassanin [3]), one sample from the CO of Colombia, and the sequence of a topotype of *M*. *rufina* (from Pichincha, Ecuador). This latter haplogroup received moderate and strong support in the ML and BI analyses, respectively, whereas the sister relationship between haplogroups A and B received non-neglible support only in the ML analysis. One sequence from Ecuador and another from Colombia were recovered as successive sisters to the clade formed by haplogroups A and B (always with either moderate or strong support; Fig 5).

None of the genetic distances calculated were particularly high. The mean *p*-distances within haplogroups A (from the CM; Fig 5) and B were 2.0% and 1.7%, respectively. The distances between haplogroups A and B were 3.0% and 3.1% for the *p*- and K2P-corrected metrics, respectively. With respect to a group containing all sequences from specimens from west of the Táchira Depression, haplogroup A showed distances of 2.6% and 2.7% for the *p*- and K2P-corrected metrics, respectively; the mean *p*-distance within the former group was 1.8%.

### Ecological Niche Modeling and Climatic Suitability in the Táchira Depression

**Fig 6:**
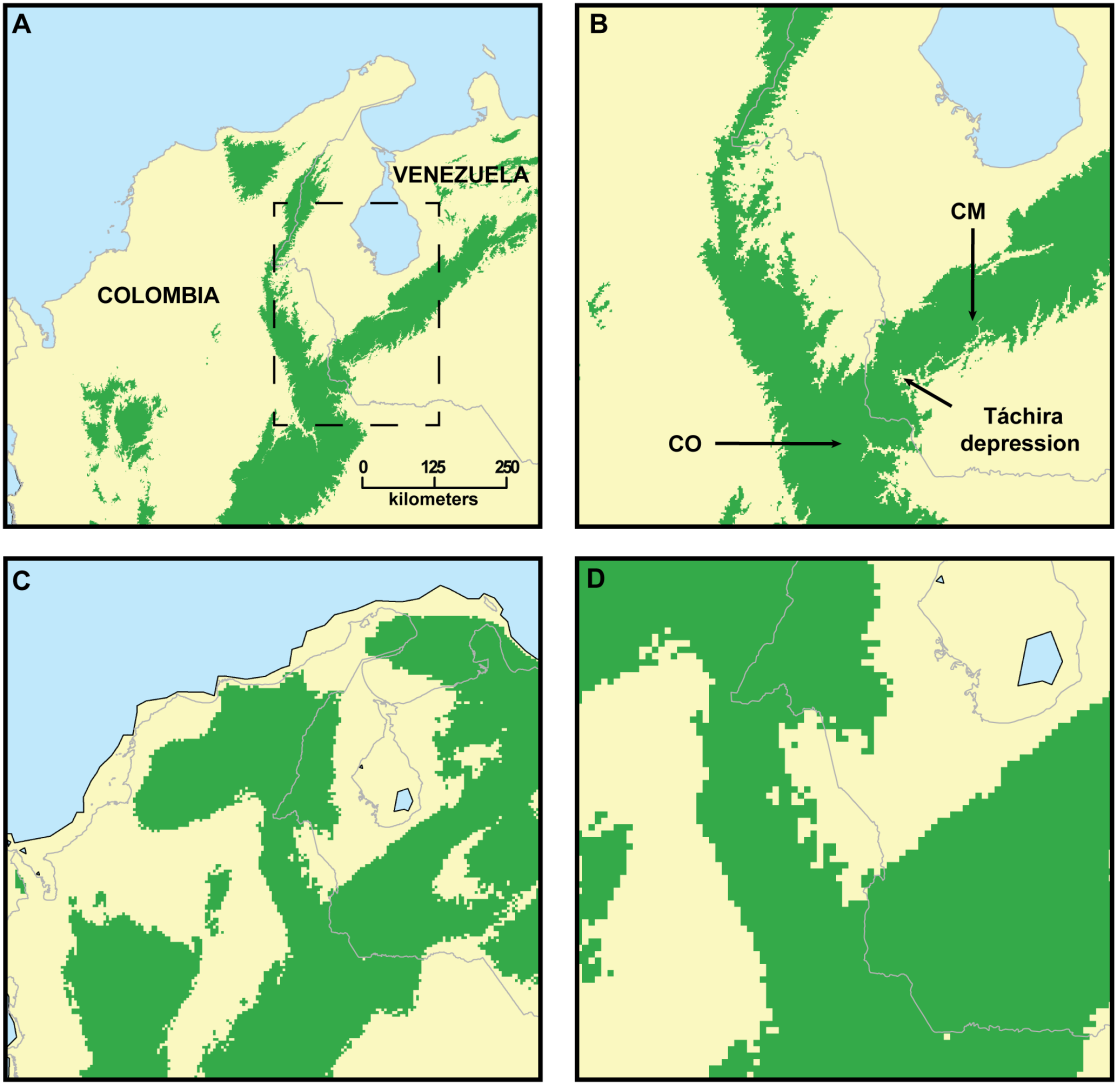
Abiotically suitable areas for *Mazama rufina* as predicted by ecological niche modeling analyses. In green are areas predicted to be suitable under current climate conditions (A, B) and during the Last Glacial Maximum (C, D). Dotted line in A circumscribes the region shown in the close up panels (B, D) and contains the Táchira Depression. Both sets of models indicate extensive suitable conditions in the area of the Táchira Depression, suggesting long term and continuous habitat connectivity between the Cordillera Oriental and Cordillera de Mérida. The Minimum Training Presence threshold of Maxent was used to convert continuous values of predicted suitability into a binary prediction, which classifies each pixel of the image (map) as suitable or unsuitable.

Ecological Niche Models (ENMs) indicate that the Táchira Depression does not represent a current climatic barrier to dispersal of *Mazama rufina* and did not during the cooler, drier climates of the Last Glacial Maximum (LGM; Fig 6; for results of tuning see S1 Table. **Results from tuning experiments using ENMeval**). At the Minimum Training Presence (MTP) threshold the model projected to current climatic conditions indicated suitable conditions in the entire Táchira Depression. Under past conditions, suitable conditions existed across on an even more extensive area, including sites of much lower elevation. Both model projections onto datasets of LGM climate conditions (CCSM in Fig 6; MIROC-ESM in S1 Fig **Maxent models of abiotically suitable areas for *Mazama rufina* projected onto estimated climatic conditions of the Last Glacial Maximum.**) indicated that the Táchira Depression contained suitable conditions for *M*. *rufina* at that time; however, the two projections differed in their overall predictions. While there is still much work to be done on assessing the effects of alternate paleo-climate reconstructions on ENMs, at least one study has shown that models based on CCSM tend to agree more strongly with independent data [76]. Inspections of clamping maps and MESS surfaces did not indicate that any climatic conditions in the projection region (and time period) were outside of the range of conditions used to calibrate the models.

## Discussion

### *Mazama bricenii* is not a valid taxon

Results of our analyses based on morphological and molecular data do not support recognition of *Mazama bricenii* as a valid taxon at either the species or subspecies level, and should be regarded as a junior synonym of *Mazama rufina*. Specimens from the CM cannot be distinguished from the rest of the specimens based on cranial morphology. Principal component analysis (PCA) is an ordination technique that allows detection of morphometric discontinuities in size, proportion, or both among taxa or geographic groups in which such discontinuities exist, e.g., [77, 78, 79, 80, 81]; however, the wide overlap in specimen scores on the first two components evince lack of morphometric discontinuity. In addition, the fact that the qualitative cranial traits that we evaluated did not show consistent differences among geographic groups is congruent with our conclusion that specimens from regions where previous authors have identified specimens as “*M*. *bricenii”* are cranially indistinguishable from *Mazama rufina*. This lack of distinctiveness is in contrast to the cases of the only two other known medium to large-sized mammals considered endemic to the CM, the Mérida mountain coati, *Nasuella meridensis* [79], and the Mérida páramo deer, *Odocoileus lasiotis* (see page 59 of [25]), both of which show substantial cranial differences with respect to their congeners in the Colombian Andes.

Phylogenetic analyses of molecular data also failed to support the validity of *Mazama bricenii*. Because we used a fast-evolving marker, if samples from the CM—or from both the CM and the CO—represented a taxon distinct from *M*. *rufina*, then we might expect them to be recovered as a reciprocally monophyletic haplogroup with respect to *M*. *rufina*. However, the two sequences from the CM were embedded within the shallow diversity of *M*. *rufina*, rendering that taxon paraphyletic. It is likely that future studies using markers with even faster mutation rates (e.g., the mitochondrial control region) could recover the samples from the CM—or from the CM and the CO—as a reciprocally monophyletic haplogroup with respect to *M*. *rufina*; however, even considering that potential topology, recognition of “*bricenii*” at the species level would be compromised by its lack of morphological diagnosabilty [82].

The genetic distances between geographically defined groups attributable to *Mazama bricenii* and *M*. *rufina* (ca. 3%) scarcely exceeds within-group variation (ca. 2%). This pattern does not suggest the presence of more than a single species among analyzed populations. Very few studies have reported genetic distances calculated from CYTB sequences between putative sister species of deer. Several of these studies instead reported within-group distance ranges for all of their focal species rather than a matrix of pairwise comparisons. Despite scarcity of data to compare with, it seems clear that CYTB genetic distances between recovered haplogroups A and B (Fig 5) are not particularly high (ca. 3%) relative to reported ranges of interspecific distances in other cervids, e.g. among various Neotropical cervids (including taxa in multiple genera; 8.6–13.9% for K2P-corrected distances) [2], among various species of muntjac, *Muntiacus* (6.5–8.7% K2P-corrected distances) [83], or between the closely related species *Cervus elaphus* and *C*. *canadensis* (5.7% uncorrected *p*-distance) [84].

In summary, our results from analyses of morphological and molecular data demonstrate that populations previously referred by authors as “*Mazama bricenii”* or “*Mazama rufina bricenii”* do not merit taxonomic recognition, and populations previously referred to by these names should be regarded as *Mazama rufina*. Although sufficient evidence supporting the validity of *M*. *bricenii* has never been published, the long-lived notion that populations from the CM were a valid species endemic to that cordillera was widely accepted simply because the Táchira Depression has been regarded as an important barrier to dispersal between the CO of Colombia and the CM. Statements like the following, by influential mammalogist J. A. Allen (page 529 of [13]), illustrate this way of thinking:

“*For example*, Mazama rufina *of Mount Pichincha in Ecuador and* M. brincenii *of the paramo of the Sierra de Merida in Venezuela so closely resemble each other in size*, *in coloration*, *and in the peculiar character of the pelage*, *that if their known ranges were contiguous they would naturally be regarded as local forms of a single species*, *but their wide separation by regions of much lower elevation and very different climatic conditions renders improbable any continuous distribution and consequent geographical intergradation*.”

### Biogeographic significance of the Táchira Depression and mammalian endemism in the CM

The warm and dry climatic conditions present in the Táchira Depression now separate cooler and more mesic habitat types (cloud forest and páramo) in the CM from similar habitats in the CO of Colombia. Because of its distinctive climate and large area, the Táchira Depression could indeed represent an important barrier to dispersal for species with lower vagility and strictly restricted to cloud forest, páramo, or both. The suspicion that the Táchira Depression represented a barrier for dispersal of *Mazama* “*bricenii*” (= *M*. *rufina*) very likely biased the view that regarded it as a valid species, implying the assumption that the “species” may had differentiated in isolation in the CM. Nevertheless, specimen-based research has shown that the Táchira Depression should not be assumed to be a barrier for species not restricted to cool and mesic habitats typical of high elevations of the northern Andes. Examples include a heteromyid rodent (*Heteromys australis*) and a didelphid marsupial (*Marmosa waterhousei*), which were recorded in the CM until recently [85, 86]. Both species were known to occur in the Colombian CO [87, 88], but the current climatic conditions of the Táchira Depression made their presence in the CM highly unexpected. Although *H*. *australis* inhabits very mesic evergreen forests up to ca. 2500 m in elevation, and *M*. *waterhousei* inhabits humid lowland and mountain forests from 50 m to 1100 m, neither of these species is restricted to habitat types with low temperatures [85, 88, 89]. As previously suggested [85, 86], finding these species in the CM indicates that the Táchira Depression was not always a barrier for them. During the LGM, about 26,500–20,000 years before present [90], the altitudinal zonation of mountains was affected with the descent of upper vegetation belts to lower elevations. This phenomenon could have led to a connection of cool and mesic habitat between the CO and the CM over the area currently occupied by the warm and dry Táchira Depression [85, 86]. Congruent with this possibility, the projection of the ENM of *M*. *rufina* onto estimated climatic conditions of the Táchira Depression during the LGM revealed that suitable climatic conditions for the species existed then. The ENM analyses show that, even at present, suitable climatic conditions (at least minimally) exist for *M*. *rufina* in the Táchira Depression (Fig 6). Similarly, results of ENM analyses in a previous study predicted suitable climatic conditions at present in the Táchira Depression for another species, *Nephelomys meridensis* [65], supposedly endemic to the CM (cf [91]).

The most recent isolation of cool and mesic habitat in the CM should not have initiated until the beginning of the current interglacial period ca. 15,000–12,000 years before present [92, 93], or perhaps even more recently. Thus, even for species restricted to cloud forest and páramo, the period of time of isolation may have been too short for populations to differentiate. In the case of *Mazama rufina*, suitable climatic conditions are still present in the Táchira Depression, therefore, populations in the CM may have never become fully isolated. This hypothesis may apply also to other members of the cordillera’s biota. To explore this possibility, we revisited the list of mammals that have been considered endemic to the CM [30]. We first corrected previous omissions of species’ records reported in the literature, and then updated the list of endemics based on recently reported records and taxonomic changes. Four species were implicated in this verification process: one didelphid marsupial (*Gracilinanus dryas*), one echimyid rodent (*Olallamys edax*), and two sigmodontine rodents (*Nephelomys meridensis* and *Neusticomys mussoi*). All of these species occur in Colombia as well as in the CM as documented in previously overlooked or unavailable literature [91, 94, 95, 96]. If the original colonization of the CM by these species, or their ancestors, was from the CO—which seems plausible given the pattern of nestedness observed in other mountain systems in the region [97]—then the fact that these species are not endemic to the CM signifies that Táchira Depression did not represent a barrier to their dispersal and distribution, as previously implied [30]. In addition, another sigmodontine rodent, *Oecomys flavicans*, previously considered to be endemic to the CM, has recently been reported for the non-Andean Serranía de San Luis in northwestern Venezuela [97]. One addition to the list of CM endemics is the procyonid *Nasuella meridensis*, which was not recognized as a valid species until recently [26]. Our overview of literature identified six species currently considered endemic to the CM. These include one soricid, *Cryptotis meridensis* [28, 98], one procyonid, *N*. *meridensis* [26], one cervid, *Odocoileus lasiotis* [4, 25], and three sigmodontine rodents, *Aepeomys reigi* [99], *Thomasomys vestitus* [100, 101, 102], and an undescribed sigmodontine rodent of the genus *Nephelomys* [103, 104].

Results from our ENM analyses, and the fact that several species once thought to be endemic to the CM are actually present in the CO, indicate that the Táchira Depression should not be invoked *a priori* as a barrier that could have promoted isolation and subsequent differentiation of taxa in the CM, unless the following two criteria are met: logically, that (1) evidence (e.g. data indicating morphological and/or molecular distinctiveness) exists suggesting that the putative taxon endemic to the CM is distinctive from congeneric populations from other regions, particularly from the nearby Andes of Colombia, including the Venezuelan portion of the CO (El Tamá); and that (2) the putative endemic taxon is currently restricted to either cloud forest, páramo, or both. The latter is proposed because mammals present in habitats with either warm or dry (or both) climatic conditions have been consistently found both to the east and west of the Táchira Depression (i.e., present in the CM as well as in the CO).

Of the six mammal species currently known only from the CM, most do not satisfy these criteria. The unnamed species of *Nephelomys* does not match the second criterion (i.e. it is not restricted to cloud forest or páramo). This species is only known from a single locality in seasonal forest at an elevation of 1100 m [104] nearby Pregonero, a town in southwestern CM. The remaining five species are only known from cloud forest or páramo habitat, or both; however, two of them, *Thomasomys vestitus* and *Odocoileus lasiotis*, remain to be compellingly compared to congeners in the Colombian Andes, especially in the CO. In the case of *T*. *vestitus*, karyological data have been gathered, but did not reveal substantial differences with respect to several other species of *Thomasomys* that would otherwise support its taxonomic status. In the case of *O*. *lasiotis*, its taxonomic status has been assessed by comparison of qualitative and quantitative morphological data with respect to other Venezuelan and North American populations of *Odocoileus*, but similar comparisons with respect to populations from the Colombian Andes remain to be conducted. It is noteworthy, however, that examination of a small number of specimens revealed substantial differences in pelage and cranial morphology between *O*. *lasiotis* and specimens from the Colombian Andes, and a plausible hypothesis for the isolation of *O*. *lasiotis* in the páramos of the CM has been postulated [25]. Finally, three species, *Aepeomys reigi*, *Cryptotis meridensis*, and *Nasuella meridensis*, match both of our proposed criteria for regarding a taxon as endemic to the CM with confidence. These species are currently restricted to cloud forest and páramo above ca. 1600 m in elevation—in the case of *A*. *reigi* between 1600 and 3230 m [99], *C*. *meridensis* 1670–3950 m, and *N*. *meridensis* 1980–4000 m [26, 28, 38, 98, 105, 106, 107]. Thus, our review suggests that the CM is likely home to true mammalian endemism, comprising species restricted to highest elevation mesic habitats, but that the level of endemism in these mountains is probably not as substantial as previous reviews have suggested. With the exception of three species, all other species currently considered endemic to the CM may be better regarded more tentatively as putative endemics until further studies are undertaken to assess their taxonomic status and distribution, both of which are essential components for planning for their effective of conservation.

## Supporting Information

S1 Fig Maxent models of abiotically suitable areas for *Mazama rufina* projected onto estimated climatic conditions of the Last Glacial Maximum. A: projection onto the Community Climate System Model dataset (CCSM4); B: projection onto the Model for Interdisciplinary Research on Climate dataset (MIROC). While in both cases suitable conditions are predicted in the Táchira Depression, the shape and strength of the prediction varies.
(TIF)

S1 File Gazetteer, specimens examined, and GenBank accession numbers. (DOCX)

S2 File Primer pairs used for amplification and sequencing of the CYTB gene. (DOCX)

S1 Table Results from tuning experiments using ENMeval. (DOCX)
