# Supplementary figures and images for: The Taxonomic Status of Mazama bricenii and the Significance of the Táchira Depression for Mammalian Endemism in the Cordillera de Mérida, Venezuela
Source: PLoS One. 2015 Jun 29;10(6):e0129113. doi: 10.1371/journal.pone.0129113 (PMC4488270; doi:10.1371/journal.pone.0129113)

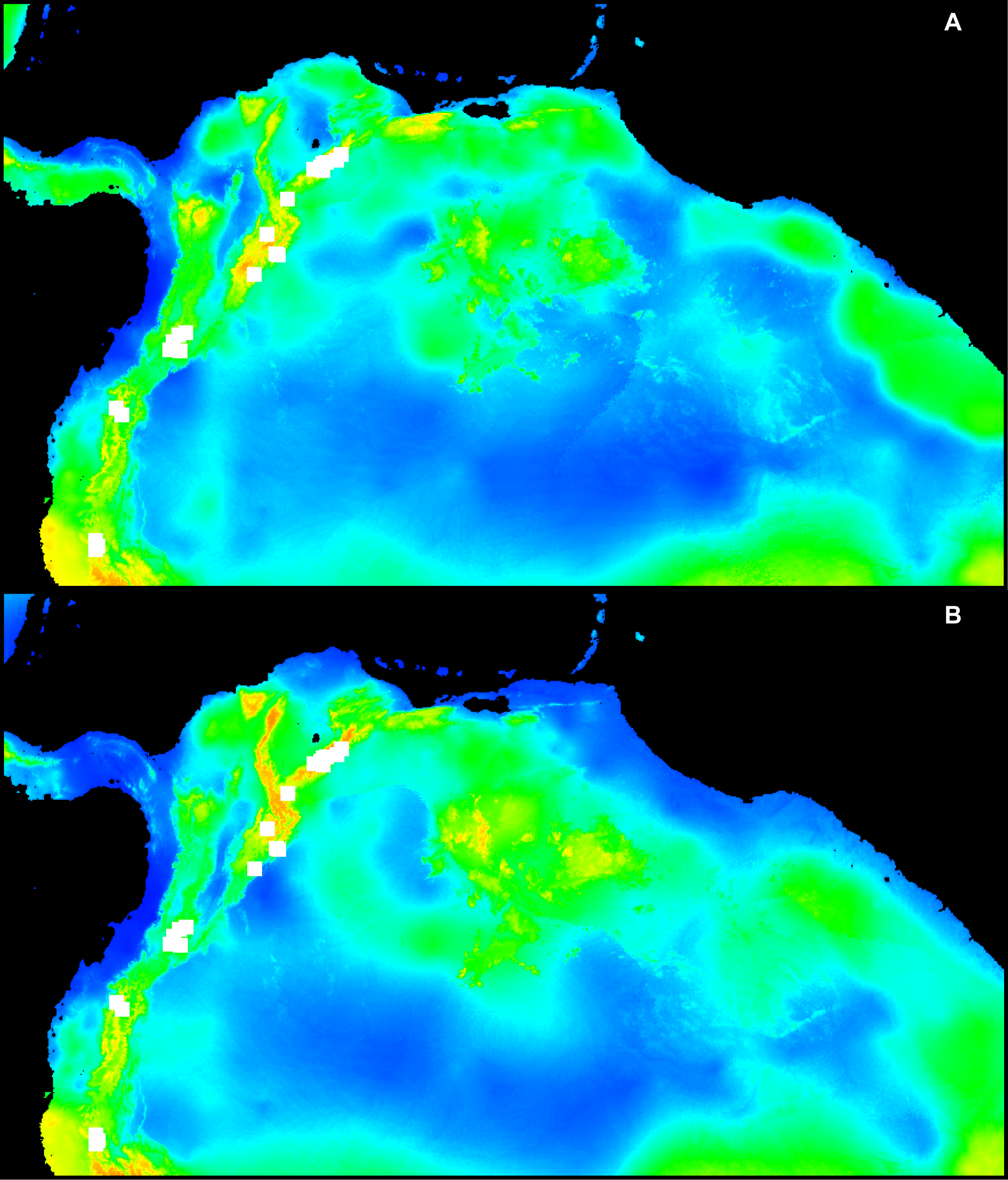

Supplement: S1 Fig — A: projection onto the Community Climate System Model dataset (CCSM4); B: projection onto the Model for Interdisciplinary Research on Climate dataset (MIROC). While in both cases suitable conditions are predicted in the Táchira Depression, the shape and strength of the prediction varies. (TIF) [file pone.0129113.s001.tif]
