# Supplementary material for: The Taxonomic Status of Mazama bricenii and the Significance of the Táchira Depression for Mammalian Endemism in the Cordillera de Mérida, Venezuela
Source: PLoS One. 2015 Jun 29;10(6):e0129113. doi: 10.1371/journal.pone.0129113 (PMC4488270; doi:10.1371/journal.pone.0129113)
Supplement: S1 File — (DOCX) [file pone.0129113.s002.docx]

**S1 File. Gazetteer, specimens examined, and GenBank accession numbers.—**Below is a list of localities, specimens of *Mazama* from the northern Andes whose associated data were employed in various analyses, and GenBank accession numbers for sequences of CYTB generated by us. Information in brackets was obtained from sources other than the collector and, where applicable, is followed by a citation to the source (see also References in the main text of the article). Localities are numbered to correspond to Figure 3 and are arranged alphabetically first by country, and then either by department (for Colombia), province (Ecuador), regions (Peru), or state (Venezuela). For each entry, boldface type indicates the place’s name or description to which geographic coordinates correspond. For each locality we list corresponding museum specimens catalog numbers. We examined all specimens reported here with exception when otherwise noted. Specimens examined are housed in the following institutions: American Museum of Natural History (AMNH; New York); Colección de Mamíferos, Instituto de Ciencias Naturales Universidad Nacional de Colombia (ICN, Bogotá, Colombia); Colección de Vertebrados de la Universidad de los Andes (CVULA; Mérida, Venezuela); Estación Biológica de Rancho Grande (EBRG; Aragua, Venezuela); Field Museum of Natural History (FMNH; Chicago); Museo de Biología de la Universidad Central de Venezuela (MBUCV; Distrito Capital, Venezuela); National Museum of Natural History (USNM; Washington DC). Specimens reported in the literature [34, 42] and whose locality data were used for ecological niche modeling analyses are housed in the scientific collection of the Centro de Ornitología y Biodiversidad (CORBIDI; Lima, Peru); Museo de Ciencias de la Universidad Símon Bolívar (MCUSB; Miranda, Venezuela); Natural History Museum of Basel (NHMB; Basel, Switzerland).

COLOMBIA

Boyaca

1. **Pajarito**, arriba de Corinto, entre Vadohondo y Corinto, 2000 m (5.4° N, 72.67° W; [108]), ICN 2992, 2994.

2. Pueblo Viejo [**Aquitania**], vereda Sisvaca, Las Vegas, 2700 m (5.45° N, 72.75° W; [108]), ICN 149.

Cauca

3. **Gabriel Lopez**, (2968 m, 2.48° N, 76.3° W; [108]), FMNH 88491.

4. **Malvasá**, (3000–3500 m, 2.57° N, 76.07° W; [5]), FMNH 88493 (GenBank accession number: KR107040).

5. **Paletara**, 3000 m (2.22° N, 76.50° W; [108]), FMNH 85824.

6. Santa Rosa, **Laguna Cusiyaco**, (3540 m; 1.92° N, 76.60° W; [108]), ICN 506.

7. Valle de Las Papas, 3000 m (1.93° N, 76.65° W; [108] coordinates correspond to **Páramo de las Papas** at the indicated elevation], FMNH 88492.

Cundinamarca

8. **Guasca, Río Balcones**, 3000 m (4.67° N, 73.55° W; [109]), USNM 70563 (GenBank accession number: KR107038).

9. **Parque Nacional Chingaza** (4.58° N, 73.73° W; [108]). Locality description provided by Manuel Ruiz-García (in litt.), who also indicated that the individual was captured and released; no voucher specimen available, but molecular data are available for this individual (GenBank NC020721; [see 3]).

Huila

10. **San Agustín**, (1630 m; 1.88° N, 76.27° W; [108]), FMNH 70560.

Santander

11. **Encino**, 6.14° N, 73.10° W. ICN 16407.

ECUADOR

Pichincha

12. **Volcán Pichincha**, occidente, [Mt.; see 110] Pichincha (0.17° S, 78.60° W; [108]), FMNH 36471, 44334, 44335 (GenBank accession number: KR107037). This locality also includes “Pichincha”, which is the locality of specimen AMNH 66741 (GenBank accession number: KR107039).

13. Cañon **Río Pita**, 2956 m (9700 ft; 0.42° S, 78.40° W; [111] coordinates correspond to **Río Pita at indicated elevation**), AMNH 66742.

PERU

Cajamarca

14. **Cruz Chiquita** (2600 m; 5.32° S, 79.32° W; [42]), CORBIDI 0022 (not examined; reported by Barrio [42]).

15. **Pajonal** (2450 m; 5.28° S, 79.27° W; [42]), CORBIDI 0021 (not examined; reported by Barrio [42]).

16. **Tabaconas** (1790 m; 5.32° S, 79.28° W; [42]), CORBIDI 0020, 0024 (not examined; reported by Barrio [42]).

17. **Lagunas Arreviatadas** (3250 m; 5.23° S, 79.28° W; [42]), CORBIDI 0016 (not examined; reported by Barrio [42]).

Piura

18. **Cerro Las Cuevas** (2000 m; 5.03° S, 79.35° W; [42]), CORBIDI 0017 (not examined; reported by Barrio [42]).

19. **Shumaya** (2115 m; 5.35° S, 79.35° W; [42]], CORBIDI 0023 (not examined; reported by Barrio [42]).

VENEZUELA

Barinas

20. **El Celoso** (8.85° N, 70.55° W; [112]), NHMB Z11488 (not examined; reported by Dietrich [34]).

Mérida

21. **Cerca (arriba) de Lagunillas, vía La Trampa**, 1200 m, 8.52° N, 71.40° W, CVULA I-8559.

22. **Chorros de Milla, 5 km N Mérida**, 1850 m (8.63° N, 71.15° W; [108]), CVULA I-2657.

23. Finca Las Mesas, **San Jacinto, 4 km S Mérida**, 1500 m (8.58° N, 71.08° W; [108] coordinates correspond to the surroundings of San Jacinto at the indicated elevation), CVULA I-3086.

24. La Campana, 3 km en la vía hacia Barinas (1510 m sensu Dietrich [34]; 8.88° N, 70.63° W; [108] coordinates correspond to **indicated elevation on the road between Santo Domingo to Barinas, at ca. 3 km W of the political border between the Mérida and Barinas states**), MBUCV I-5054.

25. **La Culata**, 3000 m (8.75° N, 71.08° W; [113]), FMNH 20197 (GenBank accession number: KR107036).

26. **Los Nevados**, 3000 m [2000 m recorded in museum catalogue] (8.47° N, 71.07° W; 113]), FMNH 20196 (GenBank accession number: KR107035).

27. **Mérida**, (1641 m; 8.60° N, 71.13° W; [113]), USNM 172982.

28. **Qda. [=Quebrada] Chorros de Milla, 3 km N Mérida**, 1900 m (8.63° N, 71.15° W; 108]), CVULA I-6786.

29. **Vía Misintá-Mucuchíes, cerca de estación de INPRADEM**, 3100 m, 8.77° N, 70.92° W, CVULA I-8561.

30. Vuelta del Burro, **arriba de Lagunillas**. (1000–1300 m; 8.50° N, 71.40° W; [108]), CVULA D-166.

Táchira

31. **Cerro Las Copas**, 2700 m (7.45° N, 72.35° W; [108]), EBRG 2224.

Trujillo

32. Las Cosinas, **4 km S of Niquitao**, 3180 m (9.07° N, 70.4° W; [108]), MCUSB I-1158 (not examined; reported by Dietrich [34]).

References cited

108. GE (Google Earth). 2013. Google Earth 7.1.2.2041 Available at https://www.google.com/earth/

109. Paynter RA Jr. Ornithological gazetteer of Colombia. 2nd ed. Cambridge: Museum of Comparative Zoology, Harvard University. 1997

110. Allen JA. List of mammals collected for the American Museum in Ecuador by William B. Richardson, 1912–1913.Bull Amer Mus Nat Hist. 1916;XXXV: 113–125.

111. IGM (Instituto Geográfico Militar). Píntag, Provincia de Pichincha, Ecuador. CT-ÑIII-D1, 3992-IV. 1972.

112. MISL (Mapas e imágenes de satélite de Latinoamérica) (2015). http://mapasamerica.dices.net Retrived 10 January 2015.

113. Paynter RA Jr. Ornithological gazetteer of Venezuela. Cambridge: Museum of Comparative Zoology, Harvard University; 1982.
