# Supplementary material for: The Taxonomic Status of Mazama bricenii and the Significance of the Táchira Depression for Mammalian Endemism in the Cordillera de Mérida, Venezuela
Source: PLoS One. 2015 Jun 29;10(6):e0129113. doi: 10.1371/journal.pone.0129113 (PMC4488270; doi:10.1371/journal.pone.0129113)
Supplement: S2 File — (DOCX) [file pone.0129113.s003.docx]

**S2 File. Primer pairs used for amplification and sequencing of the CYTB gene.** We designed all primers listed below, with exception of LGL765 forward (Bickham et al. 1995), LGL766 reverse (Bickham et al. 2004), IDMAZ224L, and IDMAZH (González et al. 2009).

| Primer name | Primer sequence |
| --- | --- |
| LGL765 forward | 5´ GAAAAACCAYCGTTGTWATTCAACT |
| CYTBPerR2 | 5´ TCAGCCGTAGTTGACGTCTC |
| IDMAZ224L | 5´ CATCCGACACAATAACAGCA |
| IDMAZH | 5´ TCCTACGAATGCTGTGGCTA |
| GrayCYTB369L | 5´ TTGGAGTAATTCTCCTATTCACAGT |
| GrayCYTB478H | 5´ AATCGGGTTAGGGTTGCTTT |
| GrayCYTB503L | 5´ TAGTTGAATGAATCTGAGGAGGCT |
| GrayCYTB649H | 5´ GTAGGGGTGGAATGGGATTT |
| GrayCYTB613L | 5´ CTTTTTCTCCACGAAACAGGA |
| GrayRedCYTB764H | 5´ GTGGGTTWGCTGGGGTGTAG |
|  |  |
| GrayCYTB756L | 5´ GCACCAGACCTACTCGGAGA |
| RedCYTB898H | 5´ ATGAAATAGGGGTATAAGGATTAGAA |
|  |  |
| GrayCYTB756L | 5´ GCACCAGACCTACTCGGAGA |
| GrayCYTB901H | 5´ TGTGGAGTAGAGGTATGAGAATCA |
|  |  |
| GrayCYTB867L | 5´ CGATCAATCCCAAATAAACTAGG |
| GrayRedCYTB992H | 5´ TCCGATTCATGTRAGTGTTAGTARG |
| GrayRedCYTB981L | 5´ CAGCCAATGTCTMTTCTGARY |
| GrayCYTB1114H | 5´ TTCATTTTAGGAGGTTGTTTTCGA |
|  |  |
| GrayRedCYTB981L | 5´ CAGCCAATGTCTMTTCTGARY |
| GrayCYTB1114H | 5´ TTCATTTTAGGAGGTTGTTTTCGA |
| GrayRedCYTB981L | 5´ CAGCCAATGTCTMTTCTGARY |
| LGL766 reverse | 5´ GTTTAATTAGAATYTYAGCTTTGGG |

References cited

Bickham JW, Wood CC, Patton JC. Biogeographic implications of cytochrome-*b* sequences and allozymes in Sockeye (*Oncorhynchus nerka*). J Hered. 1995;86: 140–144.

Bickham JW, Patton JC, Schlitter DA, Rautenbach IL, Honeycutt RL. Molecular phylogenetics, karyotypic diversity, and partition of the genus *Myotis* (Chiroptera: Vespertilionidae). Mol Phylogenet Evol. 2004;33: 333–338.

González S, Maldonado JE, Ortega J, Talarico AC, Bidegaray-Batista L, Garcia JE, Duarte JM. Identification of the endangered small red brocket deer (*Mazama bororo*) using noninvasive genetic techniques (Mammalia; Cervidae). Mol Ecol Res. 2009;9: 754–758.
