# Supplementary material for: The Taxonomic Status of Mazama bricenii and the Significance of the Táchira Depression for Mammalian Endemism in the Cordillera de Mérida, Venezuela
Source: PLoS One. 2015 Jun 29;10(6):e0129113. doi: 10.1371/journal.pone.0129113 (PMC4488270; doi:10.1371/journal.pone.0129113)
Supplement: S1 Table — (DOCX) [file pone.0129113.s004.docx]

**S1 Table. Results from tuning experiments using ENMeval.** Matrix of evaluation criteria sorted by AICc, the optimality criterion used for model tuning in this study. The combination of feature class and regularization multiplier with the lowest AICc was considered the ‘best’ model and used for final model calibration and all subsequent analysis; here the best combination of settings were Linear and Quadratic and regularization multiplier = 3. The default settings (Linear, Quadratic, and Hinge; regularization = 1) led to models that were substantially worse. Mean omission rate was calculated using the minimum training presence threshold.

| Feature  Class | Regularization Multiplier | Mean  Test AUC | Mean  AUC DIFF | Mean  Omission Rate | AICc | Model Parameters |
| --- | --- | --- | --- | --- | --- | --- |
| LQ | 3 | 0.7939 | 0.0219 | 0.0833 | 557.315288 | 4 |
| LQH | 3 | 0.7939 | 0.0455 | 0.1750 | 557.315295 | 4 |
| LQH | 3.5 | 0.7945 | 0.0309 | 0.1750 | 558.060530 | 4 |
| LQ | 3.5 | 0.7913 | 0.0222 | 0.0833 | 558.060535 | 4 |
| LQH | 4 | 0.7933 | 0.0224 | 0.0917 | 558.921589 | 4 |
| LQ | 4 | 0.7902 | 0.0224 | 0.0417 | 558.921591 | 4 |
| L | 2 | 0.7960 | 0.0242 | 0.0833 | 559.501899 | 4 |
| LQH | 4.5 | 0.7954 | 0.0226 | 0.0417 | 559.899430 | 4 |
| LQ | 4.5 | 0.7902 | 0.0226 | 0.0417 | 559.899453 | 4 |
| L | 3.5 | 0.7865 | 0.0247 | 0.0833 | 560.048947 | 3 |
| L | 2.5 | 0.7913 | 0.0232 | 0.0833 | 560.462867 | 4 |
| LQH | 6 | 0.7969 | 0.0238 | 0.0417 | 560.526986 | 3 |
| LQ | 6 | 0.7888 | 0.0238 | 0.0417 | 560.526986 | 3 |
| LQH | 5 | 0.7982 | 0.0231 | 0.0417 | 560.996116 | 4 |
| LQ | 5 | 0.7898 | 0.0231 | 0.0417 | 560.996116 | 4 |
| L | 4 | 0.7896 | 0.0247 | 0.0833 | 561.648277 | 3 |
| L | 3 | 0.7857 | 0.0245 | 0.0833 | 561.653986 | 4 |
| LQH | 5.5 | 0.7976 | 0.0235 | 0.0417 | 562.214434 | 4 |
| LQ | 5.5 | 0.7892 | 0.0235 | 0.0417 | 562.214434 | 4 |
| LQH | 2.5 | 0.7931 | 0.0551 | 0.2333 | 562.513507 | 6 |
| L | 4.5 | 0.6308 | 0.0247 | 0.0833 | 563.490087 | 3 |
| LQ | 1 | 0.7999 | 0.0446 | 0.1750 | 563.774490 | 7 |
| LQ | 2.5 | 0.7974 | 0.0216 | 0.0833 | 563.844769 | 6 |
| L | 1 | 0.7996 | 0.0354 | 0.0917 | 563.905335 | 6 |
| L | 1.5 | 0.7993 | 0.0319 | 0.0417 | 565.258699 | 6 |
| L | 5 | 0.6308 | 0.0247 | 0.0833 | 565.588765 | 3 |
| L | 6 | 0.5000 | 0.0000 | 0.0000 | 566.611515 | 2 |
| LQ | 2 | 0.7995 | 0.0305 | 0.0833 | 566.665826 | 7 |
| L | 5.5 | 0.6206 | 0.0328 | 0.0833 | 567.961546 | 3 |
| LQH | 2 | 0.7909 | 0.0709 | 0.2333 | 569.589397 | 8 |
| LQ | 1.5 | 0.7982 | 0.0365 | 0.1333 | 570.265477 | 8 |
| H | 6 | 0.7928 | 0.0312 | 0.0833 | 571.207394 | 5 |
| L | 0.5 | 0.7947 | 0.0517 | 0.0917 | 571.961856 | 8 |
| LQ | 0.5 | 0.8018 | 0.0534 | 0.1333 | 572.790078 | 9 |
| H | 3.5 | 0.7948 | 0.0438 | 0.1333 | 574.941041 | 8 |
| H | 4.5 | 0.8020 | 0.0286 | 0.0833 | 578.810366 | 8 |
| H | 5.5 | 0.8021 | 0.0328 | 0.0833 | 583.139824 | 8 |
| H | 5 | 0.8039 | 0.0304 | 0.0833 | 586.834245 | 9 |
| H | 4 | 0.7980 | 0.0364 | 0.0833 | 589.608700 | 10 |
| H | 2.5 | 0.7916 | 0.0610 | 0.2333 | 602.980395 | 12 |
| H | 3 | 0.7942 | 0.0551 | 0.2333 | 604.918968 | 12 |
| H | 2 | 0.7916 | 0.0695 | 0.1917 | 612.619933 | 13 |
| LQH | 1.5 | 0.7850 | 0.0954 | 0.1833 | 1417.350750 | 20 |
| H | 1.5 | 0.7831 | 0.0949 | 0.1833 | NA | 24 |
| H | 1 | 0.7793 | 0.1273 | 0.1417 | NA | 24 |
| **LQH** | **1** | **0.7784** | **0.1314** | **0.2250** | **NA** | **25** |
| H | 0.5 | 0.7764 | 0.1524 | 0.2667 | NA | 50 |
| LQH | 0.5 | 0.7708 | 0.1611 | 0.2667 | NA | 49 |
